# Supplementary material for: Comparative Physiological and Transcriptome Analysis Reveals Potential Pathways and Specific Genes Involved in Waterlogging Tolerance in Apple Rootstocks
Source: Int J Mol Sci. 2023 May 26;24(11):9298. doi: 10.3390/ijms24119298 (PMC10253570; doi:10.3390/ijms24119298)
Supplement: Supplementary file 1 [file ijms-24-09298-s001.zip › ijms-2346340-supplementary.pdf]

Supplemental Table S1. Summary of sequencing reads after filtering.

Sample	Clean reads	Clean base (bp)	Length	Q20 (%)	Q30 (%)	GC (%)
TCK1	22,722,980	6,816,894,000	150	98.1	94.9	48.8
TCK2	23,401,536	7,020,460,800	150	98.1	94.9	48.8
TCK3	27,171,856	8,151,556,800	150	98.2	95.1	48.8
TW1	28,967,961	8,690,388,300	150	97.8	94.4	48.2
TW2	24,493,539	7,348,061,700	150	98	94.6	47.8
TW3	23,119,949	6,935,984,700	150	98	94.6	48.1
HCK1	24,187,565	7,256,269,500	150	98.1	95	48.8
HCK2	25,405,327	7,621,598,100	150	97.9	94.7	48.2
HCK3	24,268,524	7,280,557,200	150	98.1	94.9	48.2
HW1	26,140,310	7,842,093,000	150	98.1	95	48.8
HW2	26,308,384	7,892,515,200	150	98.1	94.8	48.2
HW3	22,724,292	6,817,287,600	150	98.1	94.9	48.2

Supplemental Table S2. Clean reads mapped to the reference genome.

Sample	Total read pairs	Mapped reads	Uniq mapped reads	Multiple mapped reads
TCK1	22722980	17008792(74.85%)	13703479(60.31%)	3305313(14.55%)
TCK2	23401536	17518998(74.86%)	14063568(60.10%)	3455430(14.77%)
TCK3	27171856	20306014(74.73%)	16130817(59.37%)	4175197(15.37%)
TW1	28967961	21340677(73.67%)	17386306(60.02%)	3954371(13.65%)
TW2	24493539	18357165(74.95%)	15191108(62.02%)	3166057(12.93%)
TW3	23119949	17501953(75.70%)	14428342(62.41%)	3073611(13.29%)
HCK1	24187565	18366682(75.93%)	14830831(61.32%)	3535851(14.62%)
HCK2	25405327	18671853(73.50%)	15422375(60.71%)	3249478(12.79%)
HCK3	24268524	18238388(75.15%)	15093680(62.19%)	3144708(12.96%)
HW1	26140310	20507024(78.45%)	17203502(65.81%)	3303522(12.64%)
HW2	26308384	20007355(76.05%)	16809971(63.90%)	3197384(12.15%)
HW3	22724292	17300681(76.13%)	14568322(64.11%)	2732359(12.02%)

Supplemental Table S3. qRT-PCR primers used in this study.

No	Gene ID	F/R(Primer)	Sequence (5'-3')
1	MD15G1060800	Forward	ATCAAGAGCTGCAACATGAAAGG
		Reverse	CTCACCATCGCAATTAACGAACT
2	MD17G1081000	Forward	ACCATCTGTGTGTGGAGAAG
		Reverse	TCTGCGTTTGAACCAAGCC
3	MD11G1306200	Forward	CGGTGCTTTCATTGTTCTTTG
		Reverse	CTCCCGTTTCTTGACGCTTA
4	MD01G1083400	Forward	CGGAGGCATTTACTTACCACA
		Reverse	TAGTCCTTGGCTTGGAATACAC
5	MD10G1061300	Forward	GGAAAGAAACATGGCTACGACG
		Reverse	GGAGGCATTGGAACTGAGGAT
6	MD10G1121700	Forward	TTGCTGTGCCACCACTGTCT
		Reverse	TGCTTCACTCCCTCAACCTG

Supplemental Figure S1. A graphical abstract of how the data have been generated in this study.

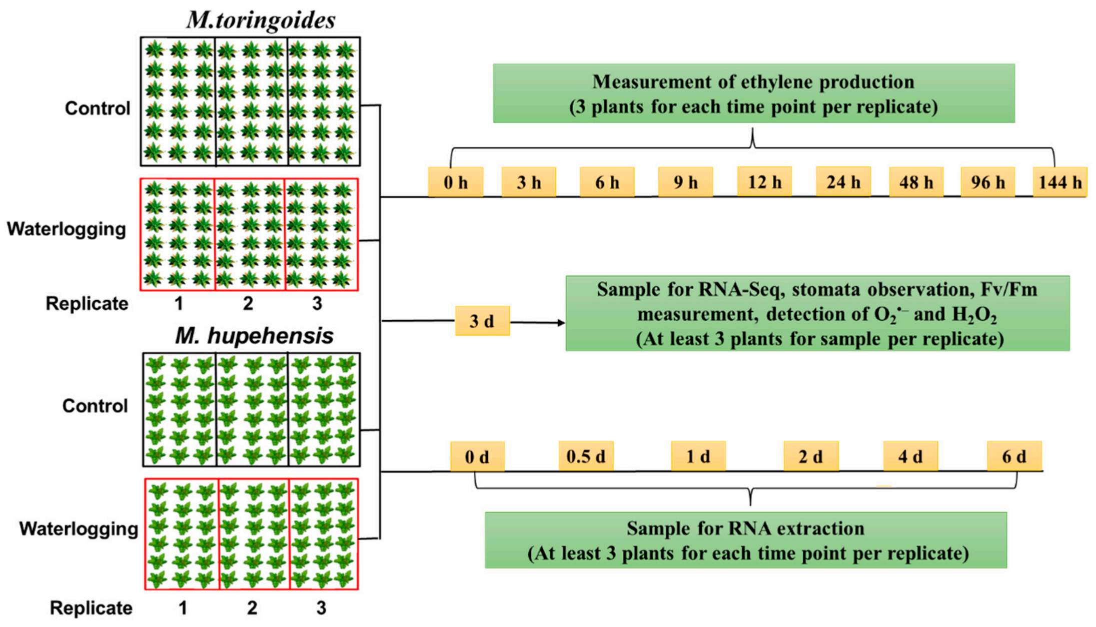

Supplemental Table S4 The information of the number of seedling sampled for each replicate in the respective experiment. Note: for measurement of ethylene, 6 seedlings were measured throughout the time points under waterlogging stress.

Experiment	Number of seedlings in each replicate	Time point	Total
ethylene	6	9	6
Fv/Fm	6	1	6
Stomata analysis	3	1	3
NBT/DAB staining	3	1	3
RNA-seq	3	1	3
RWC and EL analysis	3	6	18
RNA extraction	3	6	18
Total			57

Supplemental Figure S2. KEGG pathway enrichment analysis of DEGs identified in *M.toringoides* and *M.hupenensis* under waterlogging stress. The topmost enriched 20 KEGG pathways in the TCK-vs-TW and HCK-vs-HW comparisons were selected for display.

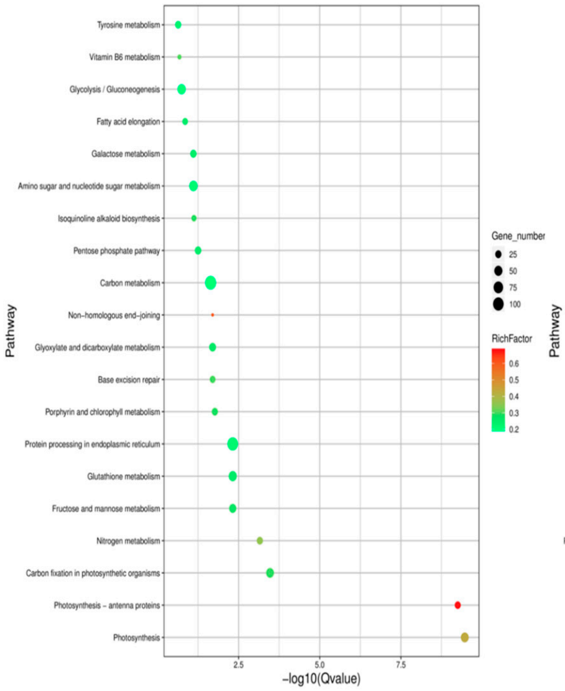

TCK-vs-TW

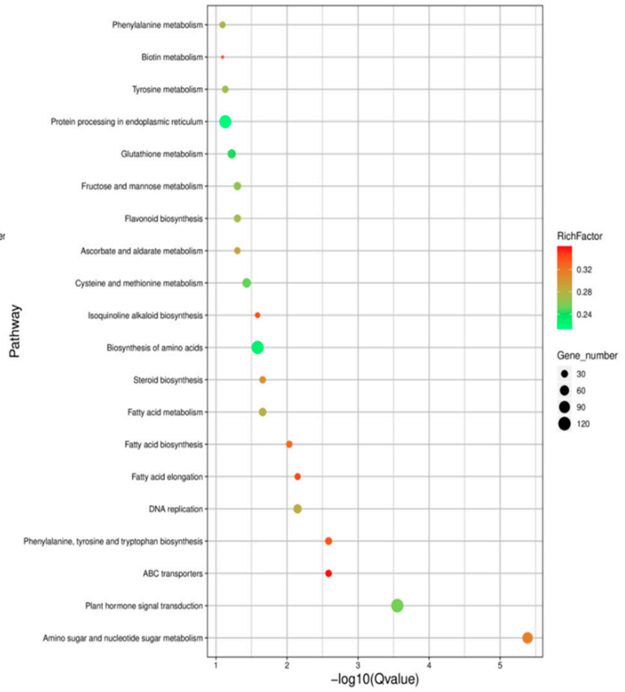

HCK-vs-HW
